# Comparative analysis of enamel mineral content and surface morphology alterations after bleaching: a study using colorimetric spectrophotometry and scanning electron microscopy

**DOI:** 10.3389/fdmed.2025.1613733

**Published:** 2025-10-31

**Authors:** Ashwini Kuruba, Geeta Ishwarappa Bolbandi, Shrikar R. Desai, Shreeshail Indi, Mohammed Mustafa, Abdullah M. Alshehri, Ali Robaian Alqahtani, Khalid K. Alanazi, Mohammed Almuhaiza, Shahad Alghannam

**Affiliations:** ^1^ Department of Conservative Dentistry and Endodontics, Rajarajeshwari Dental College and Hospital, Bangalore, India; ^2^ Department of Periodontology and Implantology, HKE’S S. Nijalingappa Institute of Dental Sciences and Research, Kalaburagi, India; ^3^ Department of Conservative Dentistry and Endodontics, Al-Badar Rural Dental College and Hospital, Kalaburagi, India; ^4^ Department of Conservative Dental Sciences, College of Dentistry, Prince Sattam Bin Abdulaziz University, Al-Kharj, Saudi Arabia; ^5^ Department of Clinical and Patient Affairs at Dental College, Prince Sattam Bin Abdulaziz University, Al-Kharj, Saudi Arabia

**Keywords:** bleaching, enamel mineral content, spectrophotometry, SEM, calcium, phosphorus, McInnes solution

## Abstract

**Background:**

Bleaching agents are widely used for aesthetic dental enhancement, but concerns remain regarding their effects on enamel mineral content and surface morphology. This study aimed to evaluate the alterations in calcium and phosphorus levels and enamel microstructure following the application of three different bleaching protocols.

**Methods:**

Ninety extracted human premolars were randomly divided into three groups (*n* = 30): Group 1 – McInnes solution, Group 2 – modified McInnes solution, and Group 3 – Pola Office bleach. Each group was subdivided for calcium, phosphorus, and scanning electron microscopy (SEM) analyses. Enamel samples were collected via microbiopsy at five time points. Calcium and phosphorus were quantified using spectrophotometric analysis, and surface changes were observed using SEM.

**Results:**

All three bleaching agents resulted in measurable mineral loss. The McInnes solution group exhibited the greatest decrease in calcium concentration, particularly at T2 (*P* = 0.001) and T4 (*P* = 0.04). SEM revealed pronounced surface alterations in this group compared to modified McInnes and Pola Office groups. Remineralization was observed after storage in artificial saliva, with partial recovery of mineral levels over 14 days.

**Conclusion:**

All bleaching protocols induced varying degrees of mineral loss and surface changes, with the McInnes solution causing the most significant effects. Modified McInnes showed relatively milder alterations, suggesting a potentially safer alternative. Post-bleaching remineralization in artificial saliva showed beneficial effects.

## 1 Introduction

The significance of cosmetic dental procedures has grown in recent years as a direct result of the modern concept of smile aesthetics. This concept places an emphasis on having teeth that are white, well-aligned, and framed by the gums and lips (1). Discoloration of the teeth that is visible to the naked eye can have a detrimental outcome on a person's life. As a result, tooth bleaching is consistently ranked as one of the highest priorities for patients who prioritize having an attractive smile (2, 3). Bleaching toothpastes and professional oral prophylaxis that involve scaling and polishing, as well as in-office bleaching, are the various ways that tooth color can be improved (4–6). Current advancements in technology have made it feasible for new aesthetic materials to exist as well as for old methods, such as tooth bleaching, to become more frequently employed (2, 7). One of the most prevalent treatments performed today in the field of cosmetic dentistry is tooth bleaching, which is a process that is widely accepted as being effective in the management of discoloured and stained teeth (6, 8). The active component of the bleaching agents that are now in use are variants of peroxide, and the amounts of each component that constitutes the bleaching agent's active component might vary. They cause the chromogens in the enamel and dentine to become more transparent, which lightens the color of the tooth as a whole (4). It is common knowledge that the application of bleaching treatment can alter the morphological appearance of mineralized deposits (9).

Because enamel is the surface stratum of the tooth, it is more vulnerable to the plethora of variables and circumstances that may be found in the oral cavity. These elements can include bacteria, acids, plaque, and food particles. Hydroxyapatite, a crystalline form of calcium phosphate, makes up the vast bulk of enamel. Hydroxyapatite is a mineral that may be found in teeth. Enamel is the substance that gives teeth their characteristic white color. In spite of the fact that it is the most resistant to demineralization of any tissue in the human body, the bone is constantly losing its minerals (10). The rate of demineralization is accelerated in acidic surroundings, and one of the most common ways this might happen is through the consumption of meals that contain both acid and sugar (11). Alternatively, saliva, antibacterial agents, and ions all contribute to the process of remineralization (such as fluoride, calcium, and phosphate). Due to the extreme fragility of the equilibrium that occurs between the processes of continual demineralization and remineralization, early carious lesions are often the outcome when this balance is disrupted for a lengthy period of time (12, 13). Changes in surface microhardness, color, and lowering of the concentration of carbonate, phosphate, or calcium in enamel may occur. These changes in tooth layers' micromorphology may be associated with the constituents of the bleach, the concentrations of those agents, the pH levels at which they are used, and the various application methods (12–17).

A number of different devices, such as “Scanning Electron and Polarized Light Microscopy”, “Micro Energy-Dispersive x-Ray Fluorescence Spectrometry”, “Fourier Transform-Raman (FT-Raman) Spectroscopy”, & “Atomic Absorption Spectroscopy (AAS)”, are commonly used to study surface changes (6).

When enamel microbiopsy is performed, the use of the method of colorimetric spectrophotometry is beneficial in quantifying the minerals that are extracted from the dental substrate without causing a significant amount of damage to the tooth structure (15). Alterations in the surface's morphology can also be seen through the use of SEM.

Recent *in vitro* and *in situ* studies have demonstrated that remineralization agents like nano-hydroxyapatite and casein phosphopeptide can effectively restore mineral content post-bleaching without compromising whitening efficacy (18, 19).

The purpose of this research was to apply the colorimetric spectrophotometry to investigate the reduction in mineral content that occurred after bleaching with three distinct types of bleaching chemicals and scanning electron microscopy to investigate the alterations that occurred on the surface of the material.

## 2 Material and methods

In this *in vitro* laboratory study, ninety (90) human extracted premolar teeth were used. These teeth were obtained from the Department of Oral and Maxillofacial Surgery of Rajarajeshwari Dental College and Hospital, Bengaluru, India. The teeth were extracted for orthodontic or periodontal purposes, were non-carious, and had no enamel or dentin defects. Following extraction, the teeth were thoroughly cleaned and disinfected before being stored in a normal saline solution until further use. The study was conducted in accordance with the Declaration of Helsinki and was approved by the Institutional Ethical Committee of Rajarajeshwari Dental College and Hospital, Bengaluru, India.

### 2.1 Materials

#### 2.1.1 Data collection and sampling technique

The three sets of specimens were randomly assigned based on the various bleaching treatments used. At the start of the procedure, specimens were kept at room temperature in a bacteriological incubator for seven days while submerged in 20 ml of artificial saliva solution.

Teeth were removed from artificial saliva for the initial enamel microbiopsy and dried using an air blast for 5 s. Based on the bleaching methods, the teeth were split into three groups:

• Group 1: 30 samples treated with McInnes solution (prepared using analytical-grade reagents from Loba Chemie, India)
• Group 2: 30 samples treated with modified McInnes solution
• Group 3: 30 samples treated with Pola Office bleach (SDI Ltd., Australia)

Each group was further divided into three subgroups:

• Subgroup A: 10 samples for calcium analysis
• Subgroup B: 10 samples for phosphorus analysis
• Subgroup C: 10 samples for SEM micro-morphological analysis

### 2.2 Enamel microbiopsy

Samples of enamel were collected using enamel microbiopsy to measure calcium and phosphorus levels. Microbiopsies were done at baseline, after first application, and before/after the second and third bleaching sessions. The sampling site was demarcated using adhesive tape with a 1.6 mm-diameter perforation. Each biopsy period used a new area.

Samples were etched with 5 µl of hydrochloric acid (HCl, Merck, India) in 70% glycerol (v/v) at 1.6 M for 20 s while gently agitated. The solution was transferred into Eppendorf tubes pre-filled with 200 µl ultrapure water. The etched surface was then rinsed with 5 ml of 70% glycerol and added to the same tubes. Centrifugation was done using a mini-spin centrifuge.

**Figure 1:**
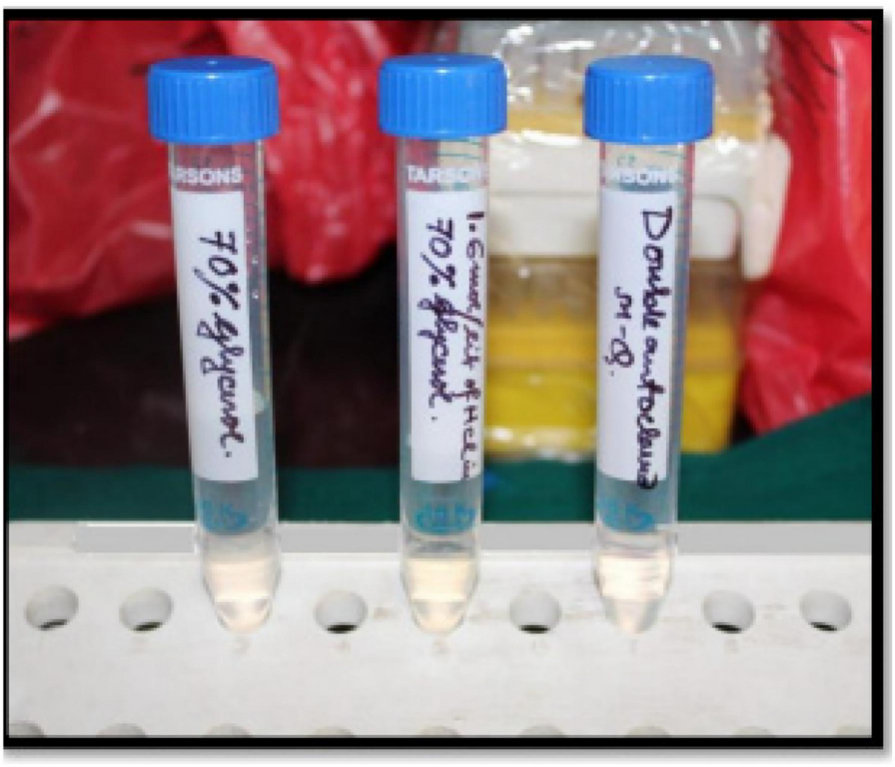
Solutions for enamel microbiopsy.

Microbiopsy time points were: baseline (Day 0), Day 7 and Day 14 before bleaching, and again on Day 7 and Day 14 post-bleaching. Samples were thawed to room temperature before chemical analysis (Figure 1).

### 2.3 Bleaching process

• **Group 1 (McInnes solution):** Prepared by mixing 1 ml of 36% HCl, 1 ml of 30% H₂O₂, and 0.2 ml of anesthetic ether in 5:5:1 ratio (all reagents from Loba Chemie, India). Applied using a cotton applicator and left on the enamel for 3–4 min, followed by rinsing with deionized water and drying.
• **Group 2 (Modified McInnes solution):** Prepared using 1 ml each of 35% H₂O₂ and 20% NaOH mixed 1:1 with diethyl ether (Qualigens, India). Application method and timing same as above.
• **Group 3 (Pola Office bleach):**
  • Liquid: 65% water, 35% H₂O₂
  • Powder: 73.26% thickeners, 26.22% catalyst, 0.5% desensitizer, 0.04% dye

**Figure 2:**
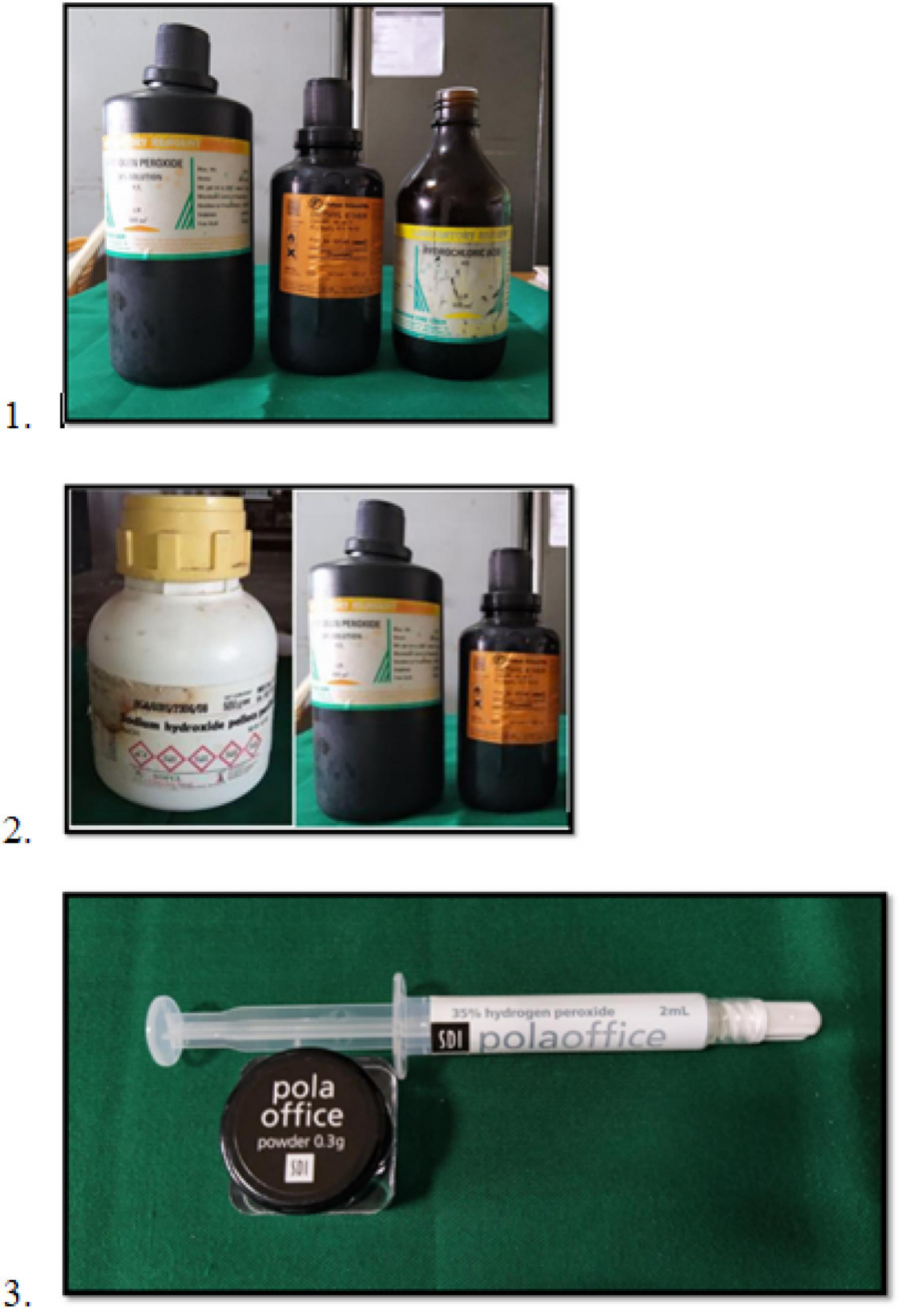
(1) McInnes bleaching solution, (2) Modified McInnes solution, (3) Pola office bleaching agent.

Contents were mixed and applied for 8 min with a brush, followed by rinsing. Microbiopsies were repeated on Days 7 and 14 (Figure 2).

### 2.4 Spectrophotometric chemical analysis

**Figure 3:**
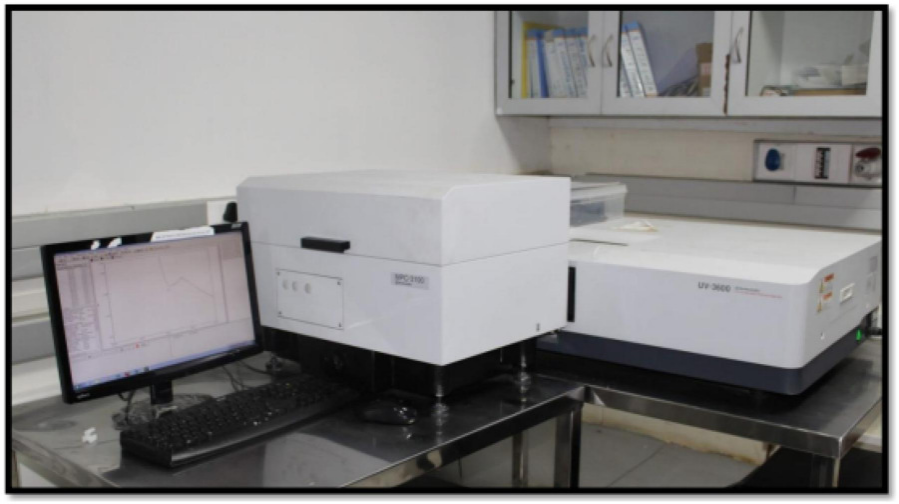
Colorimetric Spectrophotometer.

Calcium quantification was done using Arsenazo III reagent; the test solution was incubated at 37°C and absorbance was measured at 630 nm using a Shimadzu UV-1800 Spectrophotometer (Japan) (Figure 3).

Phosphorus was quantified using 50 µl of molybdic acid solution (2.5% ammonium molybdate in 4N sulfuric acid), and absorbance was read at 740 nm.

Calcium and phosphorus concentrations were expressed in µg/ml.

### 2.5 Scanning electron microscopy (SEM)

**Figure 4:**
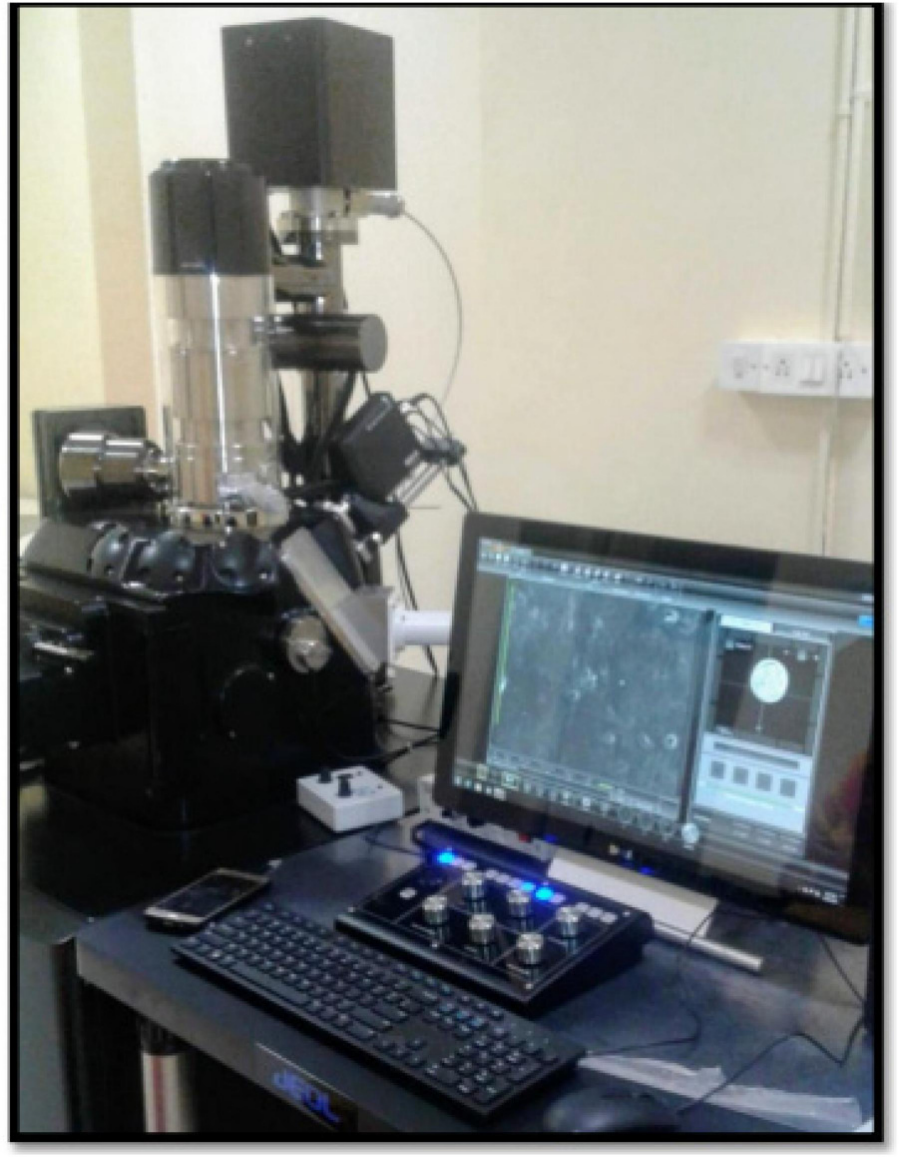
JEOL Scanning Electron Microscope (SEM).

**Figure 5:**
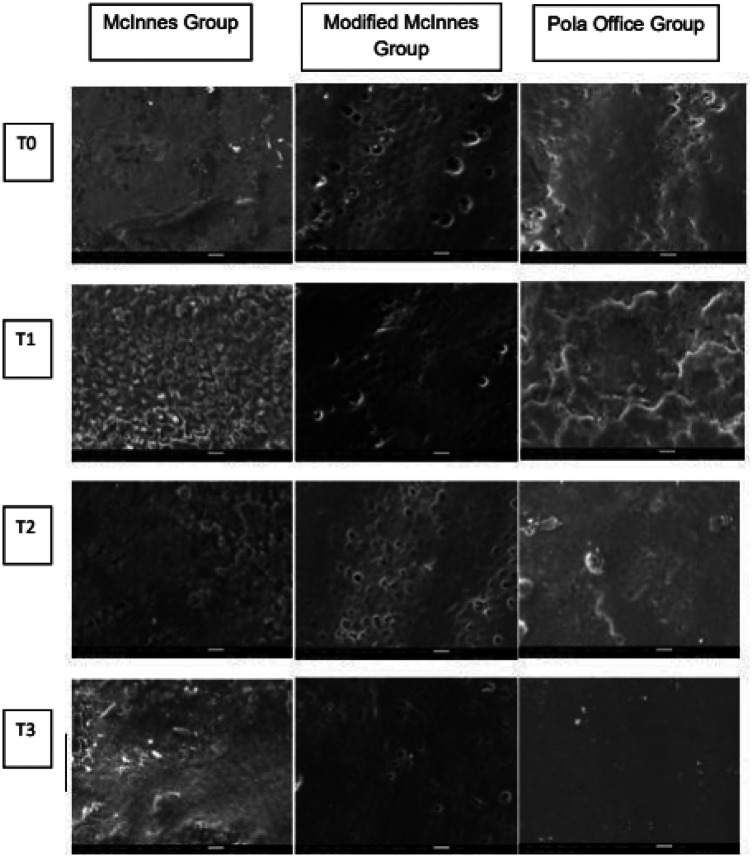
Scanning Electron Microscopic images.

Samples were observed under JEOL JSM-IT710HR SEM (Japan). After vacuum desiccation and gold sputter-coating, the specimens were examined at 1,000× magnification to assess surface changes (Figures 4, 5).

**Figure 6:**
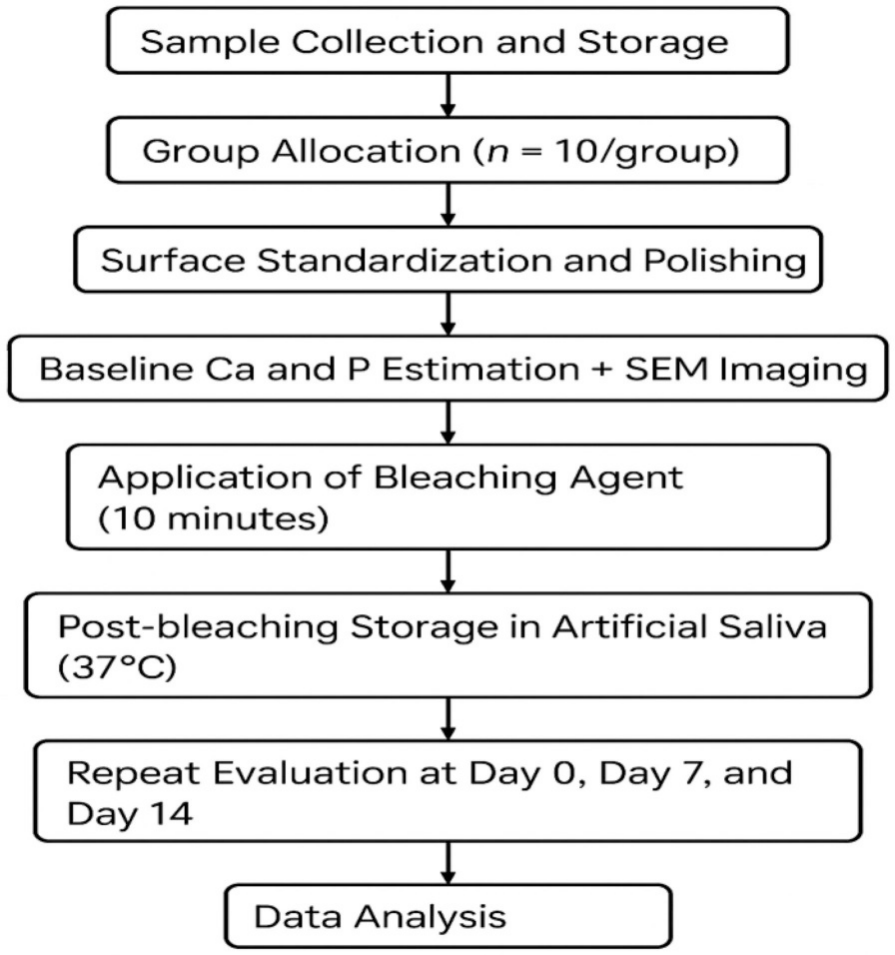
Stepwise Study Flow.

The entire stepwise study flow, right from data collection & sampling technique, enamel microbiopsy, bleaching process, spectrophotometric and SEM analysis, are shown in (Figure 6).

### 2.6 Statistical analysis

SPSS v22.0 (IBM Corp., Armonk, NY, USA) was used for data analysis.

• One-way ANOVA and Tukey's *post hoc* test: calcium between groups
• Kruskal–Wallis test: between-group comparisons of calcium
• Repeated measures ANOVA: within-group calcium comparison over time
• Friedman test followed by Wilcoxon signed-rank test: phosphorus comparisons

Significance threshold was set at *P* < 0.05.

## 3 Results

### 3.1 Calcium concentration

**Figure 7:**
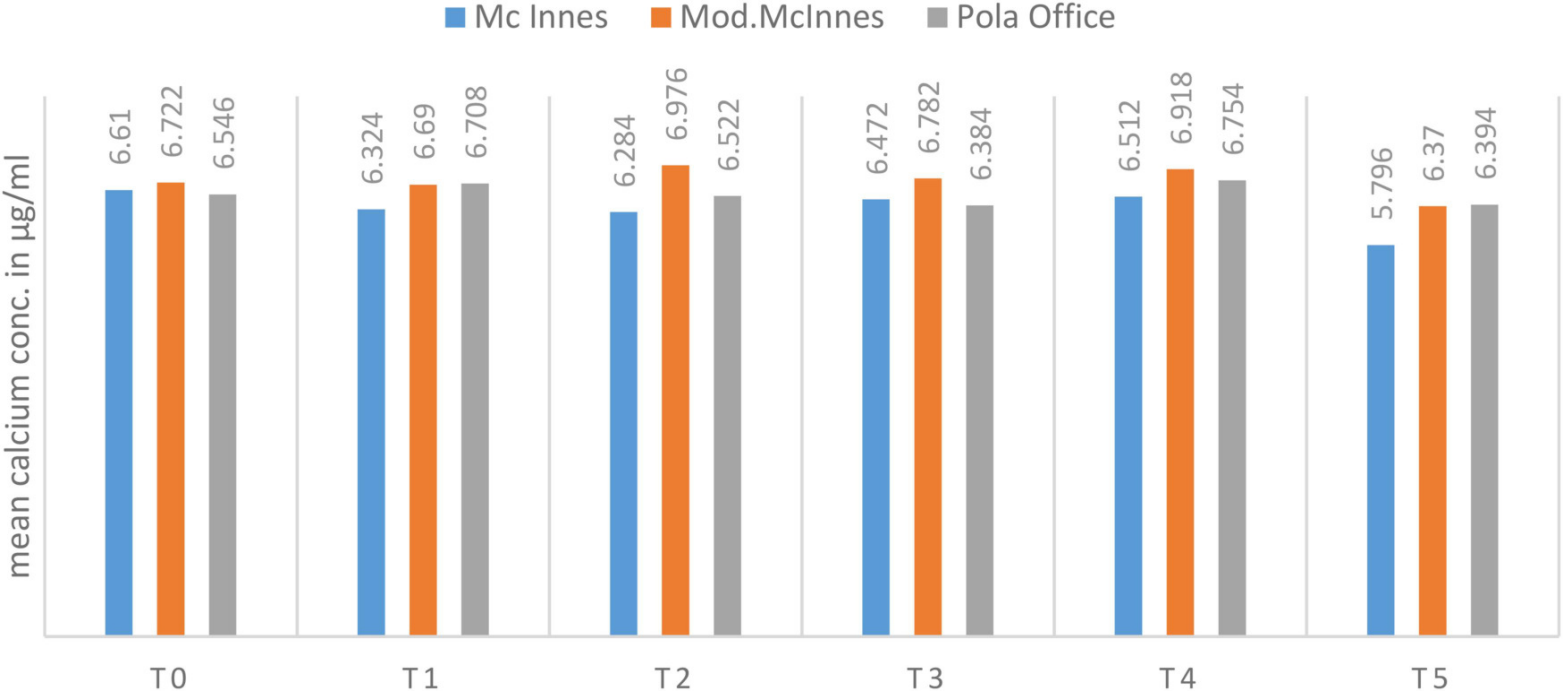
Mean calcium concentration (in µg/ml) between 03 groups at different time intervals.

A statistically significant difference in mean calcium concentration was observed among the groups at the Day 7 (T2) interval (*p* = 0.001, *η*^2^ = 0.42, large effect). The Modified McInnes group had significantly higher calcium content compared to both McInnes (*p* = 0.001) and Pola Office (*p* = 0.02). At Day 14 (T4), differences remained significant (*p* = 0.04, *η*^2^ = 0.18, moderate effect), with Modified McInnes showing better mineral preservation than McInnes (Table 1, Figure 7).

**Table 1:** Comparison of mean calcium concentration (μg/ml) among McInnes, modified McInnes, and Pola office groups at different time intervals (T0–T5) using one-way ANOVA and Tukey's *post hoc* test.

Time	McInnes		Mod. McInnes		Pola office		*p*-value	Tukey's *post hoc* Analysis		
	Mean	SD	Mean	SD	Mean	SD		M Vs MM	M vs. P	MM vs. P
T0	6.610	0.411	6.722	0.506	6.546	0.547	0.85	0.93	0.98	0.84
T1	6.324	0.246	6.690	0.344	6.708	0.304	0.12	0.17	0.15	1.00
T2	6.284	0.101	6.976	0.124	6.522	0.335	0.001*	0.001*	0.23	0.02*
T3	6.472	0.468	6.782	0.302	6.384	0.618	0.42	0.58	0.96	0.42
T4	6.512	0.211	6.918	0.232	6.754	0.230	0.04*	0.04*	0.24	0.50
T5	5.796	0.661	6.370	0.494	6.394	0.623	0.24	0.32	0.29	1.00

* *p* < 0.05 indicates statistical significance (ANOVA with Tukey's *post hoc*).

Across other time intervals, no statistically significant intergroup variation was detected (Table 1, Figure 7).

### 3.2 Phosphorus concentration

**Figure 8:**
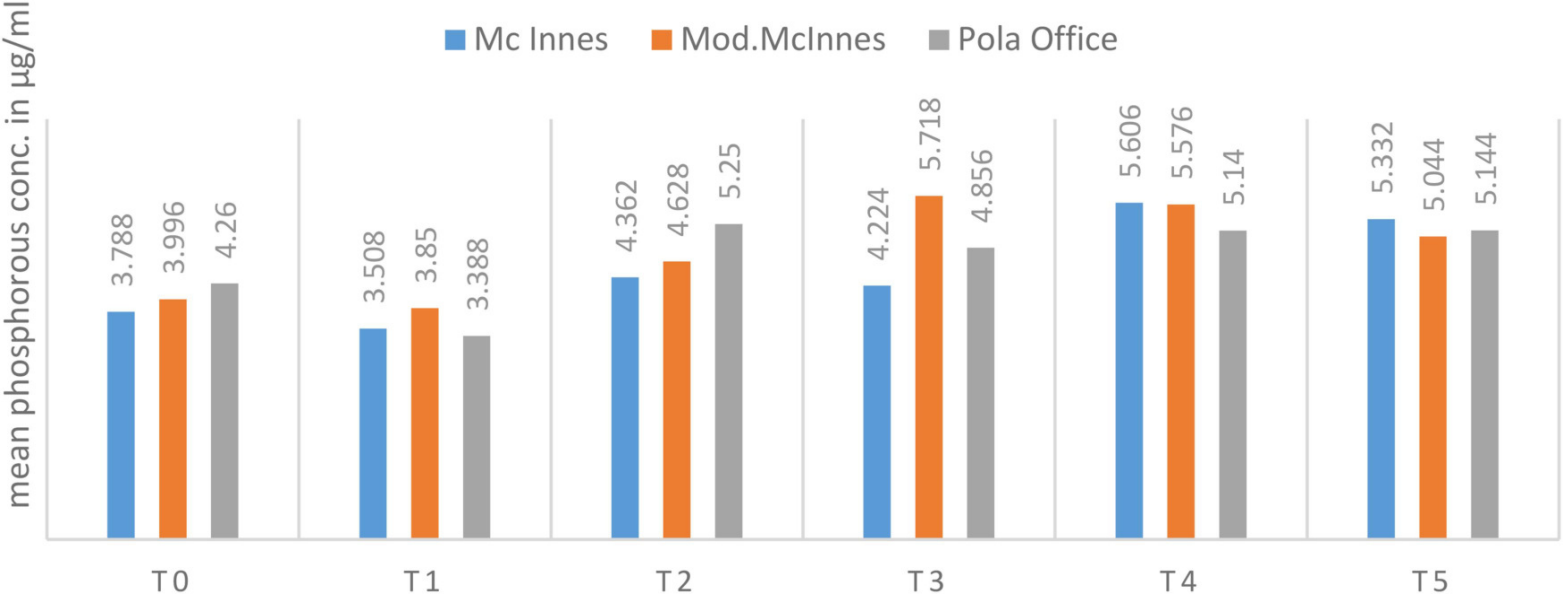
Mean phosphorous concentration (in µg/ml) between 03 groups at different time intervals.

No significant intergroup differences were found in mean phosphorus concentration at any time point (*p* > 0.05). However, variability was considerable in some subgroups, particularly the Modified McInnes group at Day 7, where standard deviations were relatively high (Table 2, Figure 8). This was attributed to small subgroup size and biological variability, and is acknowledged as a study limitation.

**Table 2:** Comparison of mean phosphorus concentration (μg/ml) among McInnes, modified McInnes, and Pola office groups across time intervals using Kruskal–Wallis test.

Time	McInnes		Mod. McInnes		Pola office		*p*-value
	Mean	SD	Mean	SD	Mean	SD	
T0	3.788	0.365	3.996	0.361	4.260	1.225	0.91
T1	3.508	0.370	3.850	0.423	3.388	0.832	0.34
T2	4.362	0.827	4.628	2.004	5.250	1.371	0.34
T3	4.224	0.850	5.718	1.519	4.856	1.046	0.33
T4	5.606	0.587	5.576	1.233	5.140	1.061	0.95
T5	5.332	1.283	5.044	2.031	5.144	1.056	0.89

### 3.3 Within-group changes in calcium

**Figure 9:**
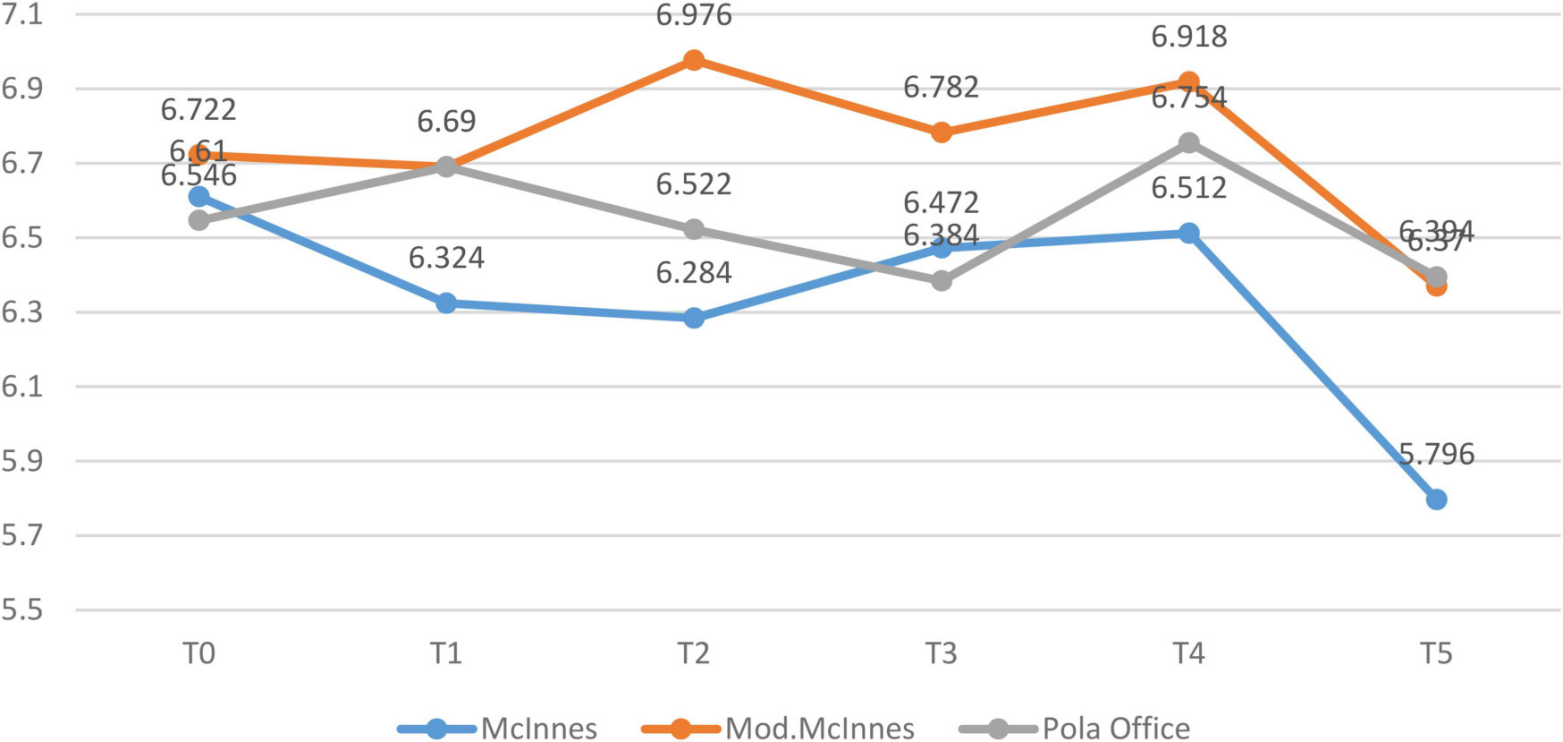
Mean calcium concentration (in µg/ml) between different time intervals in each group.

Repeated measures ANOVA revealed no significant changes in calcium levels over time in the Modified McInnes (*p* = 0.21) and Pola Office (*p* = 0.54) groups. In contrast, the McInnes group showed significant fluctuations (*p* = 0.08), with the greatest mineral loss occurring between Day 7 and Day 14 (Table 3, Figure 9).

**Table 3:** Intragroup comparison of mean calcium concentration (μg/ml) over time in each group using repeated measures ANOVA.

	McInnes		Mod. McInnes		Pola office	
	Mean	SD	Mean	SD	Mean	SD
T0	6.610	0.411	6.722	0.506	6.546	0.547
T1	6.324	0.246	6.690	0.344	6.708	0.304
T2	6.284	0.101	6.976	0.124	6.522	0.335
T3	6.472	0.468	6.782	0.302	6.384	0.618
T4	6.512	0.211	6.918	0.232	6.754	0.230
T5	5.796	0.661	6.370	0.494	6.394	0.623
*p*-value	0.08		0.21		0.54	

### 3.4 Within-group changes in phosphorus

**Figure 10:**
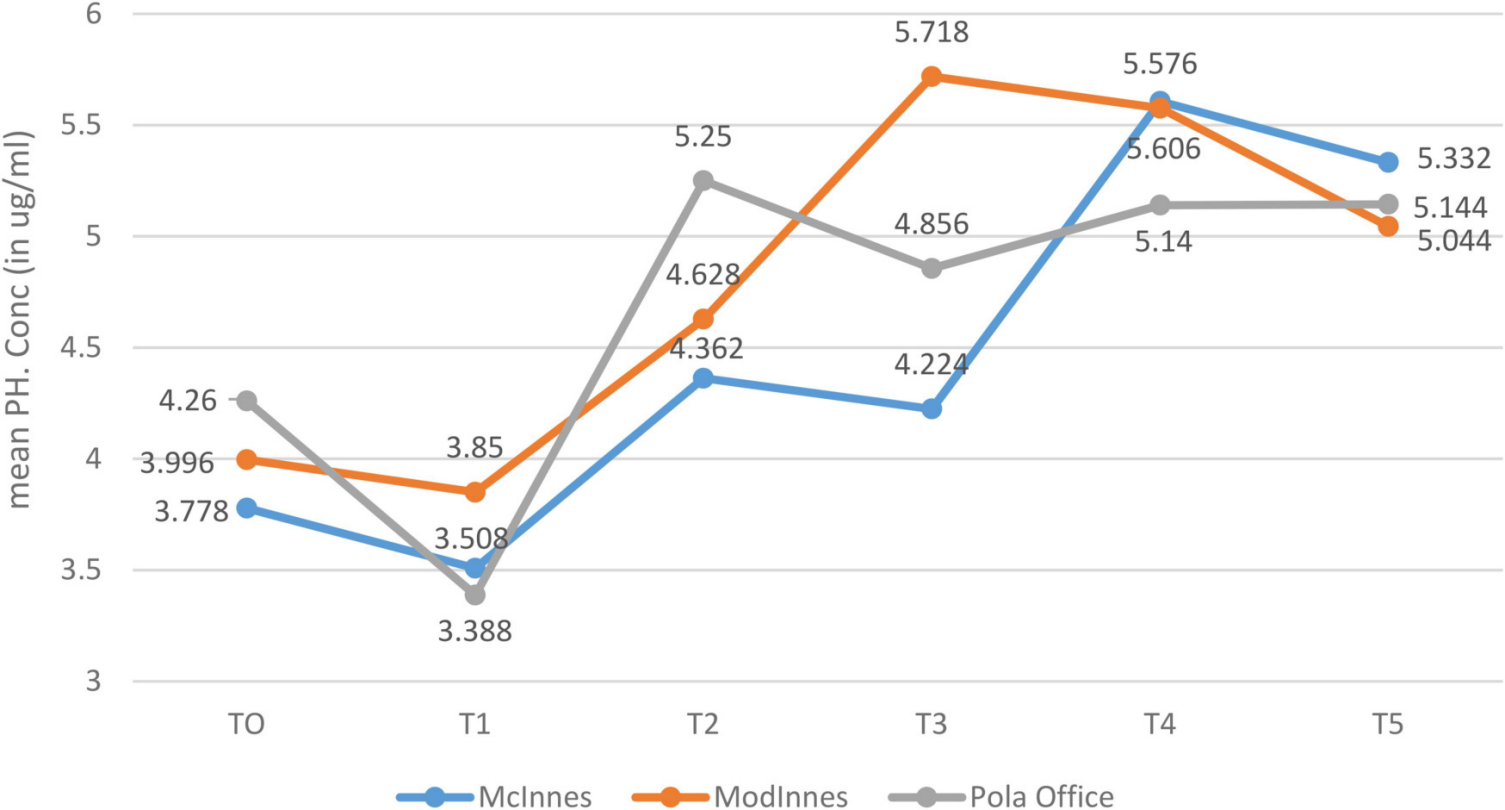
Mean phosphorous concentration (in µg/ml) between different time intervals in each group.

Within-group analysis demonstrated a significant difference in mean phosphorus concentration across time in the McInnes group (*p* = 0.007, Friedman's test, r = 0.31, moderate effect). *Post hoc* comparisons indicated that Day 14 (T4) values were significantly higher than baseline (T0) and earlier intervals (*p* = 0.04). However, no significant changes were observed in Modified McInnes (*p* = 0.08) or Pola Office (*p* = 0.52) groups (Table 4, Figure 10).

**Table 4:** Intragroup comparison of mean phosphorus concentration (μg/ml) over time in each group using Friedman's test.

Time	McInnes		Mod. McInnes		Pola Office	
	Mean	SD	Mean	SD	Mean	SD
T0	3.788	0.365	3.996	0.361	4.260	1.225
T1	3.508	0.370	3.850	0.423	3.388	0.832
T2	4.362	0.827	4.628	2.004	5.250	1.371
T3	4.224	0.850	5.718	1.519	4.856	1.046
T4	5.606	0.587	5.576	1.233	5.140	1.061
T5	5.332	1.283	5.044	2.031	5.144	1.056
*p*-value	0.007*		0.08		0.52	

* Statistically significant.

### 3.5 *Post hoc* comparisons in McInnes group

Wilcoxon signed-rank *post hoc* tests revealed multiple significant differences between time intervals in the McInnes group, confirming greater fluctuations in phosphorus concentrations (Table 5).

**Table 5:** *Post hoc* Wilcoxon signed-rank test for multiple comparisons of phosphorus concentration between time points in McInnes group.

(I) Time	(J) Time	Mean Diff. (I–J)	95% CI for Diff.		*p*-value
			Lower	Upper	
T0	T1	0.280	−1.598	2.158	0.35
	T2	−0.574	−2.648	1.500	0.23
	T3	−0.436	−3.226	2.354	0.69
	T4	−1.818	−3.900	0.264	0.04*
	T5	−1.544	−5.250	2.162	0.08
T1	T2	−0.854	−3.765	2.057	0.04*
	T3	−0.716	−2.865	1.433	0.14
	T4	−2.098	−3.844	−0.352	0.04*
	T5	−1.824	−5.177	1.529	0.07
T2	T3	0.138	−1.935	2.211	0.35
	T4	−1.244	−2.779	0.291	0.04*
	T5	−0.970	−3.818	1.878	0.23
T3	T4	−1.382	−2.298	−0.466	0.04*
	T5	−1.108	−2.686	0.470	0.04*
T4	T5	0.274	−2.057	2.605	0.23

* Statistically significant.

### 3.6 SEM observations

SEM analysis revealed distinct differences in enamel morphology (Figures 1–5).

• **McInnes group:** severe surface deterioration, including microporosities, prism disintegration, and irregularities.
• **Modified McInnes group:** comparatively smoother surfaces, with fewer microcracks and better prism preservation.
• **Pola Office group:** moderate surface alterations with visible porosities, but less severe than McInnes.

These findings supported the quantitative mineral analysis, with Modified McInnes showing superior preservation of enamel structure.

#### 3.6.1 Tables and figures cited

• Table 1: Comparison of mean calcium concentration (μg/ml) between groups at different time intervals.
  *Footnote: *p* < 0.05 indicates statistical significance (ANOVA with Tukey's *post hoc*).
• Table 2: Comparison of mean phosphorus concentration (μg/ml) between groups (Kruskal–Wallis test).
  *Footnote: High SDs reflect biological variability and small subgroup size*.
• Table 3: Within-group calcium concentration across intervals (Repeated measures ANOVA).
• Table 4: Within-group phosphorus concentration across intervals (Friedman's test).
• Table 5: *Post hoc* comparisons of phosphorus concentration within McInnes group (Wilcoxon signed-rank).
• Figures 1–5: Experimental setup, bleaching agents, colorimeter, SEM machine, and representative SEM images.
• Figures 7–10: Visual representation of calcium and phosphorus changes over time.

## 4 Discussion

The practice of bleaching, which dates back more than a century, has been around for quite some time (18–20). As a result of the recent surge in popularity of cosmetic dentistry, many individuals are now opting for bleaching procedures to enhance their dental aesthetics. When vital teeth are bleached, the enamel surface is exposed for a prolonged duration to gels rich in oxidizing agents. The exposure duration depends on the formulation used, which raises concern about potential enamel damage (21–25).

The structural integrity of organic enamel components, including collagen and proteins, can be compromised by oxidizing agents used in bleaching (22, 26, 27). Studies have reported decreased micro-tensile strength, fracture resistance, increased porosity, surface roughness, fluoride loss, mineral loss, altered Ca/P ratio, and organic matrix disruption (28, 29). These effects support the hypothesis that the chemical agents in bleaching formulations may weaken the mineralized structure of teeth (23, 30–34).

Surface microhardness assessment via the Vickers test has proven effective in evaluating enamel changes post-bleaching (23, 24, 31). Mechanical changes vary with testing parameters such as load, dwell time, and indent location (35–37).

Since demineralization was found to be localized primarily to surface enamel, spectrophotometric chemical analysis was selected as a minimally destructive tool for assessing mineral changes. Enamel structure and composition are known to vary regionally depending on its anatomical position, leading to localized differences in mineral response (24, 25, 37–39).

In this study, the enamel was segmented into three parts to independently assess calcium, phosphorus, and micro-morphological changes via SEM. The bleaching agents compared were the McInnes solution, modified McInnes solution, and Pola Office bleach. Previous studies confirm that peroxide-based bleaching agents alter the inorganic content of enamel, reducing Ca and P concentrations and disrupting enamel crystals (26).

Only a limited number of studies have specifically quantified mineral loss using the same bleaching agents as used here. Spectrophotometric analysis provides a novel, reproducible technique for enamel microbiopsy, offering a practical way to evaluate mineral composition without compromising structural integrity (18, 21).

The analysis revealed that all groups experienced a measurable reduction in mineral content post-bleaching, regardless of the agent used. Hydrogen peroxide, a common component in all three agents, acts by generating reactive oxygen species (ROS) that degrade pigmented molecules (chromophores) (30). Exposure of enamel to acidic conditions leads to dissolution of hydroxyapatite by hydrogen ions (31).

Group-wise comparisons indicated that Group 1 (McInnes solution) exhibited the greatest mineral loss, which is attributed to its content of 30% hydrochloric acid. This is consistent with findings by Tezel et al., who demonstrated enamel decalcification with HCl concentrations between 18% and 30% (31). Choi et al. similarly reported notable enamel etching within two minutes of HCl application *in vitro* (32).

To minimize HCl's erosive effect, Chen Hua Ji et al. substituted 20% sodium hydroxide (NaOH) in the modified McInnes solution (26). The modified version used a 1:1 ratio of 30% H₂O₂ and 20% NaOH, which increased the oxidative efficiency of hydrogen peroxide in alkaline media while reducing enamel damage. Their findings — that modified McInnes produces less enamel damage — align with those of the present study.

Mineral loss observed in the Pola Office group was comparable to the other two groups. Its high 35% H₂O₂ concentration results in a low pH of ∼4.5, contributing to its aggressiveness. Tezel et al. also noted that higher peroxide concentrations extract more Ca^2+^ ions, increasing enamel susceptibility (33).

After treatment, teeth were stored in artificial saliva to simulate oral conditions. Remineralization was observed over 7 and 14 days, supporting prior findings by Haleh Heshmat et al., which attribute this to phosphate and calcium ion saturation (40). This super saturation promotes both resistance to demineralization and enhances natural remineralization potential (37). However, *in vivo*, saliva alone may be insufficient, necessitating additional remineralizing agents after bleaching.

SEM analysis showed that Group 1 had the most severe surface alterations, corroborating the results of Rajesh A. G. et al., who linked extensive morphological damage to HCl (37). The findings of Ji Chen et al. on the milder effects of modified McInnes were also validated (26). In the Pola Office group, variable surface roughness and cracking were noted. These outcomes are consistent with Souza RO et al., who linked high H₂O₂ concentrations to enamel chemistry and morphology changes (30).

Both SEM and spectrophotometric findings confirm that enamel damage post-bleaching is influenced by agent type, pH, and application duration (39, 41, 42). These results align with prior enamel studies (43–45).

## 5 Limitations

This study has certain limitations that must be considered when interpreting the findings. First, it was conducted exclusively under *in vitro* conditions. Although artificial saliva was used to simulate the oral environment, it does not fully replicate the complex biological reality of the oral cavity, including dynamic salivary flow, enzymatic activity, biofilm interactions, and mechanical forces such as mastication. These factors may substantially alter enamel demineralization and remineralization *in vivo*.

Second, the sample size within each subgroup was relatively small (*n* = 10 per time point). A formal power analysis was not performed, and the limited sample size may have reduced statistical power and increased variability. This is reflected in some groups, particularly in phosphorus concentration measurements, where standard deviations were high. Consequently, there remains a risk of Type II error and overestimation of certain effects.

Third, although standardized bleaching protocols were employed, subtle operator-dependent variations and baseline differences in enamel composition between teeth cannot be fully excluded as potential confounders.

Fourth, the study assessed only short-term changes (up to 14 days) in enamel mineral content and surface morphology. Long-term effects, including the potential for progressive demineralization, were not addressed. Likewise, no patient-centered outcomes, such as hypersensitivity, esthetic improvement, or durability of bleaching, were evaluated.

Finally, the study did not investigate the protective role of post-bleaching remineralizing agents, which are clinically relevant in contemporary bleaching protocols and may mitigate enamel alterations. Future studies should include these comparisons to provide more translational evidence.

## 6 Conclusion

All three bleaching protocols tested in this study—McInnes solution, modified McInnes solution, and Pola Office bleach—caused varying degrees of enamel mineral loss and surface alteration.

Among them, the McInnes solution induced the most pronounced mineral depletion and surface roughness, likely due to its high hydrochloric acid content.

The modified McInnes solution demonstrated relatively milder effects on enamel, confirming its potential as a safer alternative.

Pola Office bleach also resulted in mineral loss, though at a level comparable to modified McInnes.

Spectrophotometric analysis and SEM imaging both proved useful in detecting these changes.

Post-bleaching remineralization in artificial saliva showed partial recovery of calcium and phosphorus levels, highlighting the importance of remineralizing protocols in clinical practice.

## Data Availability

The original contributions presented in the study are included in the article/Supplementary Material, further inquiries can be directed to the corresponding authors.
